# *ESR2* expression in subcutaneous adipose tissue is related to body fat distribution in women, and knockdown impairs preadipocyte differentiation

**DOI:** 10.1080/21623945.2022.2102116

**Published:** 2022-08-17

**Authors:** Fozia Ahmed, Susanne Hetty, Milica Vranic, Giovanni Fanni, Joel Kullberg, Maria João Pereira, Jan W Eriksson

**Affiliations:** a Department of Medical Sciences, Clinical Diabetes and Metabolism, Uppsala University, Uppsala, Sweden; b Department of Surgical Sciences, Radiology, Uppsala University, Uppsala, Sweden

**Keywords:** Adipocytes, adipogenesis, CRISPR/cas9, lipid metabolism, obesity, insulin resistance, type 2 diabetes

## Abstract

Oestrogen receptor 2 (*ESR2*) expression has been shown to be higher in subcutaneous adipose tissue (SAT) from postmenopausal compared to premenopausal women. The functional significance of altered *ESR2* expression is not fully known. This study investigates the role of *ESR2* for adipose tissue lipid and glucose metabolism. SAT biopsies were obtained from 44 female subjects with or without T2D. Gene expression of *ESR2* and markers of adipose function and metabolism was assessed. *ESR2* knockdown was performed using CRISPR/Cas9 in preadipocytes isolated from SAT of females, and differentiation rate, lipid storage, and glucose uptake were measured.

*ESR2* expression was inversely correlated with measures of central obesity and expression of some fatty acid oxidation markers, and positively correlated with lipid storage and glucose transport markers. Differentiation was reduced in *ESR2* knockdown preadipocytes. This corresponded to reduced expression of markers of differentiation and lipogenesis. Glucose uptake was reduced in knockdown adipocytes.

Our results indicate that *ESR2* deficiency in women is associated with visceral adiposity and impaired subcutaneous adipocyte differentiation as well as glucose and lipid utilization. High *ESR2* expression, as seen after menopause, could be a contributing factor to SAT expansion. This may support a possible target to promote a healthy obesity phenotype.

## 1. Introduction

Oestradiol (E2) signalling occurs primarily through the classical nuclear oestrogen receptors alpha (*ESR1*) and beta (*ESR2*), or the G protein-coupled receptor 1 (*GPER1*) [1]. Human adipose tissue expresses both *ESR1* and *ESR2*, which seem to play a role in whole-body adipose tissue metabolism [2]. Recently, we have shown that *ESR2* expression is higher in subcutaneous adipose tissue (SAT) of postmenopausal women, compared to premenopausal women [3,4]. High adipose tissue *ESR2* expression levels are associated with metabolic perturbations such as insulin resistance [5]. However, this notion derives from studies mainly using ovary-intact, female animal models, or cell lines [6]. Recently, it was reported that ESR2 signalling in female ovariectomized mice reduced adiposity and insulin resistance [7]; thus, challenging the paradigm that high ESR2 expression always confers metabolically detrimental effects and highlighting the importance of appropriate experimental models when investigating oestrogen receptors.

Adipose tissue insulin sensitivity is important for whole-body glucose metabolism [8,9]. Adipose tissue consists of several cell types such as adipocytes, preadipocytes, and immune cells. Although it is known that *ESR2* is higher in SAT of postmenopausal women compared to premenopausal women [3], the functional significance of this remains to be fully investigated, and what the adipocyte-specific effects of ESR2 in SAT are, is not known. Most studies on adipocyte metabolism and oestrogen receptors have focused on the role of *ESR1*. For instance, it has been shown that in 3T3-L1 adipocytes, E2 enhances insulin-stimulated phosphorylation of Akt and GLUT4 translocation, which is mainly mediated through *ESR1* [10]. The role of ESR2, however, on adipocyte glucose metabolism is still not clear. In addition, menopause, a state with high SAT *ESR2* expression, has been associated with changes in adipose tissue phenotype that are linked to altered lipid metabolism [11,12]. However, the link between *ESR2* expression and adipocyte lipid storage has not been thoroughly investigated and to our knowledge, there are no studies that have investigated the role of ESR2 in human preadipocyte differentiation into mature adipocytes.

In the present study, we investigate the role of *ESR2* deficiency on differentiation and glucose metabolism in preadipocytes isolated from females, using CRISPR/cas9 gene editing technology. We hypothesized that *ESR2* expression is important for preadipocyte differentiation and glucose uptake capacity.

## 2. Methods and subjects

### 2.1. Subjects

This study consists of three cohorts as described below:

*Cohort 1* included 20 female subjects (10 with T2D (age: 55 ± 9 y, BMI 32.6 ± 5.9 kg/m^2^) and 10 control subjects (age: 60 ± 9 y, BMI: 31.0 ± 4.7 kg/m^2^) matched for age and BMI). Clinical characteristics for cohort 1 are published in [13], which also included 20 male subjects.

*Cohort 2* was used to validate findings from cohort 1 and it included 9 female subjects with T2D (age: 49 ± 10 y, BMI 28.0 ± 3.0 kg/m^2^) and 6 female control subjects (age: 61 ± 7 y, BMI: 36.3 ± 3.5 kg/m^2^).

*Cohort 3* included 9 non-diabetic female subjects (age: 48 ± 23 y, BMI: 28.7 ± 5.9 g/m^2^) and their adipose samples were used for CRISPR/Cas9 experiments and assessment of protein expression.

All subjects were phenotyped by anthropometric and clinical characteristics (Table 1). Subjects arrived in the morning after an overnight fast at the clinical research unit at Uppsala University Hospital. Anthropometric measurements were performed, and fasting blood samples were taken to determine HbA1c, glucose, lipids, insulin, and C-peptide levels. Subjects with type 1 diabetes (T1D), other endocrine disorders, cancer, or other major illnesses, as well as treated with beta-adrenergic blockers, systemic glucocorticoids, and immune-modulating therapies were excluded from the study. T2D subjects were on a stable dose of metformin for at least 3 months. An oral glucose tolerance test (OGTT) for analyses of plasma glucose, insulin, and free fatty acids (FFA) was performed. Overall, there were 13 premenopausal and 31 postmenopausal women distributed across the cohorts. One subject had undergone tubal ligation. SAT samples were obtained by needle aspiration of the lower part of the abdomen after local dermal anaesthesia with lidocaine (Xylocaine, AstraZeneca). One part of adipose tissue biopsy was snap-frozen in liquid nitrogen and used for RNA sequencing (cohorts 1 and 2) for analyses of *ESR2* expression and association with obesity and insulin resistance markers, and expression of other genes related to adipocyte function. Isolated mature adipocytes were used for adipocyte size measurements and *ex vivo* analyses of glucose uptake (cohorts 1 and 2). Isolated preadipocytes were used for CRISPR/cas9 gene knockdown of *ESR2* (cohort 3). Not all subjects were used for all experiments due to limitations in adipose tissue quantity. The Regional Ethics Review Board in Uppsala (Dnr 2013/330 and Dnr 2013–183/494) and Gothenburg (Dnr 336–07) approved the studies, and all participants gave their written informed consent.

**Table 1.** Anthropometric and clinical characteristics of the participating women.

	Cohort 1	Cohort 2		Cohort 3
N	20	15		9
Type 2 Diabetes	10	9		0
Age (years)	58 ± 9	54 ± 11		48 ± 23
BMI (kg/m^2^)	31.8 ± 5.3	33.0 ± 5.3		28.7 ± 5.9
WHR	0.9 ± 0.1	0.9 ± 0.1		0.9 ± 0.1
Body fat (%)	NA	41.9 ± 7.0		39.9 ± 7.1
Plasma glucose (mmol/L)	6.6 ± 1.1	7.8 ± 2.1		5.5 ± 0.8
HbA1c (mmol/mol)	41.4 ± 5.7	50.1 ± 14.5		33.8 ± 3.9
Serum insulin (mU/L)	14.0 ± 7.3	20.3 ± 13.2		7.4 ± 8.4
HOMA-IR	4.0 ± 2.6	7.4 ± 5.1		3.8 ± 2.9
Total cholesterol (mmol/L)	5.4 ± 0.9	5.3 ± 1.3		5.4 ± 0.8
Plasma HDL-cholesterol (mmol/L)	1.3 ± 0.2	1.2 ± 0.4		1.8 ± 0.5
Plasma triglycerides (mmol/L)	1.5 ± 0.6	1.9 ± 1.1		1.3 ± 0.5
Plasma LDL-cholesterol (mmol/L)	3.4 ± 0.8	3.4 ± 1.2		3.5 ± 0.6

Data mean ± SD. Blood chemistry is fasting.

BMI: body mass index. WHR: Waist-to-hip ratio. HbA1c: glycated haemoglobin. HOMA-IR: Homoeostatic model assessment. HDL: high density lipoprotein. LDL: low density lipoprotein. NA: not applicable.

### 2.2. MRI

In cohort 1, MRI was used to assess volumes of abdominal SAT, visceral AT (VAT), and liver fat percentage as previously described [13].

### 2.3. Human preadipocyte isolation and CRISPR/Cas9 mediated gene knockdown of ESR2

Preadipocytes were isolated from the stromal vascular fraction (SVF) of SAT (cohort 3) as previously described [14]. In brief, the SVF was centrifuged at 1200 RPM for 3 min, and the pellet was resuspended in red cell lysis buffer (0.154 M NH_4_Cl, 10 mM KHCO3, and 0.1 mM EDTA, all from Sigma) and centrifuged again at 1200 RPM for 3 min. The pellet was resuspended and cultured with preadipocyte medium (Dulbecco’s modified Eagle’s medium (DMEM)/Ham’s F12, Gibco, Life Technologies, Paisley, UK) containing 10% foetal bovine serum (FBS, Thermo Fisher), 100 units/mL penicillin, and 100 g/mL streptomycin (PEST, Life Technology), 0.04 mg/mL gentamycin (Gibco), and 4.1 ng/mL basic fibroblast growth factor (bFGF) (Sigma) at 37°C until 70–80% confluent. *ESR2* gene knockdown was performed in preadipocytes (cohort 3) using a recently published method [15]. Briefly, single guide RNAs (sgRNA) were designed using the online tool https://chopchop.cbu.uib.no/ (2021–03-01) [16]. Initially, four sgRNAs were pre-screened for gene editing efficiency using the human Simpson Golabi Behmel Syndrome (SGBS) cell line, and the two most efficient guides, ESR2-G1 and ESR2-G2, were used for experiments in primary human SVF-derived preadipocytes (Supplementary Table 1 and figure S2). *ESR2* gene knockdown was performed by delivering *ESR2* sgRNAs and TrueCut Cas9 protein v2 as a ribonucleoprotein (RNP) complex using the Neon Transfection system (all from Thermo Fisher, MA, USA). Wild type cells were electroporated, but without any Cas9 or sgRNA, and served as a transfection control. A sgRNA targeting the safe harbour locus AAVS1 was used as negative control (Thermo Fisher), as previously shown [14]. After transfection, preadipocytes were cultured in DMEM/Ham’s F12 supplemented with 10% FBS without antibiotics for 48h (Thermo Fisher). After 48h, medium was changed to preadipocytes medium. Knockdown efficiency was measured by immunoblot (refer below, section 2.9) on d 14 of differentiation.

### 2.4. Human preadipocyte differentiation into mature adipocytes and cell proliferation

Human preadipocytes were differentiated for up to 14 d, as previously reported [14]. In brief, preadipocytes from passage 3 were used for gene editing, and plated for differentiation on passages 5–6 [14]. The cells were seeded at 10,000–30,0000 cells/cm^2^ into 12 well plates (Thermo Fisher) using preadipocyte media (section 2.3). On d 2 and 3, cells were fixed and stained for the nuclei using DAPI and imaged for cell proliferation. This was measured as the fold-change of number of cells between d 2 and 3 during preadipocyte growth. After cells reached 100% confluence, they were cultured for one additional day after which adipogenesis was induced by adding a differentiation cocktail (phenol-red free DMEM/Ham’s F12, 1% PEST, 100 nM human insulin (Actrapid, Novo Nordisk), 17 µM pantothenate (Sigma), 33 µM biotin (Sigma), 1 µM cortisol (Sigma), 1 µM rosiglitazone (Sigma), 250 µM 3-isobutyl-1-methylxanthine (IBMX, Sigma), 10 µg/ml transferrin human (Sigma), 2 nM 3, 3, 5-triiodo L-thyronine (T3, Sigma)) for 5 d with replacement of induction cocktail on d 3. On d 5, cells were then cultured in maintenance medium (composition is the same as the differentiation cocktail except for omitting IBMX) until d 14. Medium was replenished every 3 d. The degree of adipocyte differentiation was assessed using fluorescent staining of lipid droplets and by measuring the expression levels of differentiation markers in cells collected on d 0, and 7, and 14  post-induction.

### 2.5. Immunofluorescence staining for differentiation rate

Differentiation rate and lipid content were measured on d 7 and 14 of differentiation (n = 4), as described previously [14]. Briefly, cells were fixed with 4% formaldehyde (Histolab, Gothenburg) and stained with a combination of the fluorescent neutral lipid dye, BODIPY 493/503 (Molecular Probes, OR) and the nucleic stain Hoechst 33,342 (Invitrogen, MA, USA). Cells were imaged using the ImageXpress Pico (Molecular Devices) and the InCellis Fluorescent microscope (Bertin Instruments). Image acquisition for analysis was performed on the ImageXpress Pico, each well using a 10x magnification, and a 6 × 6 square image scan covering 20% of each well´s total area. Differentiation rate was calculated as the percentage of lipid-positive cells using the automated cell scoring software function (CellReporterXpress software), which identifies cells that either contains lipids (positive cells) or that do not contain any lipids (negative cells).

### 2.6. Gene expression

#### 2.6.1. Transcriptomics

SAT from cohort 1 and cohort 2 was used for RNAseq at Exiqon A/S [13], and Novogene [3], respectively. The data were used to determine correlations between *ESR2* and markers of lipid and glucose metabolism.

#### 2.6.2. Real-time quantitative PCR

Total RNA was extracted from whole adipose tissue using the RNeasy lipid tissue mini kit (Qiagen). Total RNA extraction from differentiating preadipocytes (d 7 and 14) (n = 4) was performed with the phenol–chloroform extraction method [17,18]. Cells were lysed with Trizol (Sigma), and chloroform (Sigma) was used for phase separation. Isopropanol and GlycoBlue (Invitrogen, ThermoFisher Scientific) were added to the aqueous phase in a new tube and incubated overnight at −20°C. On the next day, the pellet was washed 3 times with 70% ethanol and left to air dry. The concentration and purity of total RNA were measured with the Nanodrop (Thermo Scientific). cDNA was synthesized using a High-Capacity cDNA Reverse Transcriptase kit (Applied Biosystems) according to the manufacturer’s guidelines. mRNA expression was determined using TaqMan gene expression assays (Thermo Fisher), as described in Table 2. Gene expression was detected using the QuantStudio 3 sequence detection system (Applied Biosystem) and calculated using a 2^−deltaCT^. The gene expression levels were normalized to the housekeeping gene *GUSB*, and all samples were run in duplicates.

**Table 2.** TaqMan probes used for gene expression analyses.

Gene name	Abbreviation	TaqMan probe
Adiponectin	*ADIPOQ*	Hs00605917
Adipose triglyceride lipase	*ATGL, PNPL2*	Hs00982042
Acetyl-CoA Carboxylase Alpha	*ACACA*	Hs01046060
Carnitine palmitoyltransferase 1B	*CPT1B*	Hs00189258
CCAAT eenhancer binding protein alpha	*CEBPA*	Hs00269972
Fatty acid binding protein 4	*FAB4*	Hs01086177
Fatty acid synthase	*FASN*	Hs01005622
Glucose transporter 4	*GLUT4, SLC2A4*	Hs00168966
Hormone-sensitive lipase	*HSL, LIPE*	Hs00193510
Lipoprotein lipase	*LPL*	Hs00173425
Peroxisome proliferator-activated receptor gamma	*PPARG*	Hs01115513
Perilipin 1	*PLIN1*	Hs00160173

### 2.7. Glucose uptake

*Differentiated adipocytes, in vitro*. Glucose uptake was performed on d 10–14 of differentiation (n = 4) in wild type, negative control and knockdown cultures using the luminescence Glucose Uptake-GLO kit (Promega) according to the manufacturer’s instructions and as previously reported [18][14]. In brief, cells were washed 2 times with warmed PBS and then incubated for 2 h in Krebs Ringer HEPES (KRH) buffer containing 0.01% BSA (Sigma), with 5 mM glucose (Sigma), 200 nM adenosine (Sigma), and pH 7.4. Afterwards, cells were washed with KRH without glucose 2 times. To assess basal and insulin-stimulated glucose uptake, cells were incubated at 37°C for a total of 30 minutes in KRH no glucose medium without or with insulin (1000 mU/mL). The glucose analog 2-deoxy-2-glucose was added for the last 10 min of the incubation. The quantification was done as per the manufacturer’s instructions and corrected for protein.

*Mature adipocytes, ex vivo*. Glucose uptake in isolated adipocytes was performed as described previously [3,19]. In brief, incubated adipose tissue was digested using collagenase A (Roche) in a shaking water bath at 105 RMP for 1 h at 37°C in Medium 199 (Gibco, Life Technologies) supplemented with 6 mM glucose, 4% bovine serum albumin (BSA, Sigma), 150 nM adenosine (Sigma), pH = 7.4. Cell suspension was then passed through a 250 nM nylon mesh and collected into a falcon tube. Adipocytes were isolated from digestion media and washed three times at 5-min intervals in glucose-free KRH Krebs-Ringer bicarbonate medium (KRH) supplemented with 4% BSA, 150 nM adenosine, pH = 7.4. Next, adipocytes were incubated at 37°C in a shaking water bath at 65 RPM for 15 min in KRH medium without (basal), or with 25 or 1000 µU/ml of insulin (Actrapid). After 15 min, the cells were incubated with D-(U-14C) glucose (0.86 µM glucose, 0.26 mCi/mL, Perkin Elmer, Boston, USA) for 45 min. The reaction was stopped by transferring the reaction tubes onto ice, and cells were immediately separated from the media by centrifugation through silicon fluid (WR Chemicals). Radioactivity associated with cells was then measured with a scintillation counter (Radiomatic series 500TR, Perkin Elmer Analytical Instruments). Glucose uptake was expressed as clearance of medium glucose per cell. Cell number was determined following measurements of triglyceride content (Doles extraction) [20] and cell size (Axiovision, München, DE) [21].

### 2.8. Isolation of human adipocytes and stromal vascular fraction (SVF) for immunoblotting

Adipocytes and SVF were isolated from SAT (n = 3, cohort 3) for analysis of ESR2 protein expression, as described previously [22]. Half a gram (500 mg) of adipose tissue was digested as described in section 2.3. The floating adipocytes were washed four times in Hank’s medium (6 mmol/L glucose, 4% BSA, 0.15 μmol/L adenosine, pH 7.4) and were separated from the medium by filtration through dinonyl phthalate (Merck, Darmstadt, Germany). The SVF was isolated from the collagenase medium by centrifugation for 3 min at 1200 rpm and washed with PBS. The adipocytes and SVF were used for immunoblotting analysis of ESR2, as reported in section 2.9. Total protein levels of the adipocyte and SVF fractions were measured using BCA protein assay kit (Pierce, Thermo Scientific) and used to extrapolate the total ESR2 protein levels in the whole-cell extractions.

### 2.9. Immunoblotting

Total protein was isolated from adipocytes, SVF, preadipocytes and differentiated cells on d 14 of differentiation (n = 4), as previously reported [13]. In brief, cells were lysed in ice-cold lysis buffer: 25 mM Tris-HCl (Sigma), pH 7.4; 0.5 mM EGTA (Sigma); 25 mM NaCl (Sigma); 1% Nonidet P-40; 1 mM Na_3_Vo_4;_ 10 mM NaF; 100 nM okadaic acid (Alexis Biochemicals), 1X Complete protease inhibitor cocktail (Roche, Indianapolis, IN, USA) and 1 mM orthovanadate (Sigma). The lysate was vortexed and incubated on ice for 10 min and then centrifuged at 15,000 g for 15 min at 4°C. The infranatant was transferred into a new tube and saved at −80°C. The lysates were collected, and protein concentration was determined using a BCA protein assay kit (Pierce, Thermo Scientific). Proteins (10–20 µg) were separated by SDS-PAGE (5–8% gradient stain-free gels), transferred to nitrocellulose membranes and blocked with 0.05% tween-PBS (Medicago) with 5% non-fat dry milk (Biorad, Hercules). Membranes were incubated overnight with the primary antibody. The primary antibodies anti-ESR2 (1:500, Invitrogen, PPZ0506), anti-GLUT4 (1:1000, Invitrogen, Waltham, MA, RRID:AB_2191429), and β-Actin (1:1000, 4970S, Cell Signalling), were used for experiments. Membranes were then washed with 0.05% tween-PBS and incubated with appropriate horseradish peroxide conjugated anti-mouse or anti-rabbit (Cell Signalling) secondary antibody. Visualization of protein bands and stain-free blot images was then performed using enhanced chemiluminescence with a high-resolution field with the ChemiDocTM MP System (Biorad), and quantification was performed with Image Lab Software. ESR2 and GLUT4 protein signals were normalized with either B-actin or stain-free total protein measure.

### 2.10. Statistical analysis

All data are presented as mean ± SEM, unless stated otherwise. A comparison between two paired groups was made with paired t-test. Comparison between more than two paired groups was made using a one-way ANOVA with repeated measures or mixed model effects. Multiple comparisons were adjusted for false discovery rate (FDR) with Benjamini, Krieger, and Yekutieli’s method. Parametric test was performed on log-transformed data. Spearman’s correlation was used for bivariate analysis. A multilinear regression model was used to predict the impact of clinical variables on the *ESR2* associations with different adipogenic or glucose metabolism markers. ‘n’ refers to the number of separate individuals and their adipose samples used in each experimental setup. A P-value of less than 0.05 was considered statistically significant. All data were analysed using GraphPad Prism 9.0.2 or IBM SPSS version 23.

## 3. Results

### 3.1. Correlation between ESR2 expression and markers of obesity, hyperglycaemia, and insulin resistance

We found that *ESR2* expression in SAT from females was negatively correlated with weight, WHR, and VAT volume and positively correlated with basal and insulin-stimulated glucose uptake in isolated adipocytes (Table 3). This was validated in a separate cohort (cohort 2) which had corresponding correlations with obesity markers and *ex vivo* glucose uptake. In addition, hyperglycaemia and insulin resistance markers were negatively associated with *ESR2* expression (Table 3). There was a positive trend between *ESR2* expression and adipocyte size, however, this was not confirmed in the validation cohort. We assessed whether *ESR2* was differentially expressed in control and T2D and found no significant difference (data not shown). In addition, neither SAT or plasma metformin levels were associated with SAT *ESR2* expression (SAT: rho = −0.007, p = 0.989; plasma: rho = 0.003, p = 0.989). In addition, VAT and WHR were the strongest predictors of *ESR2* mRNA expression (VAT: p = 0.053, WHR: p = 0.054), after adjustment for T2D. These associations were also analyzed in male subjects (clinical characteristics of a male cohort from [13] are shown in Supplementary Table 2), and no significant associations were found.

**Table 3.** Correlations between *ESR2* mRNA expression and markers of obesity and insulin resistance in human adipose tissue.

	Cohort 1		Cohort 2	
	n = 20		n = 15	
	Rho	*P*-value	Rho	*P*-value
*Clinical characteristics*				
Age	0.165	0.486	0.025	0.929
BMI	−0.212	0.369	−0.354	0.196
Weight	*−0.396*	*0.087*	**−0.625**	0.013*
WHR	**−0.496**	0.026*	−0.296	0.283
Body fat %	NA		−0.293	0.289
VAT volume	**−0.489**	0.040*	NA	
SAT volume	−0.252	0.314	NA	
VAT/SAT ratio	−0.204	0.415	NA	
Liver fat %	0.016	0.951	NA	
*Hyperglycaemia and insulin resistance markers*				
HbA1c	−0.075	0.754	**−0.562**	0.029*
HOMA-IR	*−0.380*	*0.099*	*−0.502*	*0.056*
Fasting glucose	−0.167	0.480	*−0.506*	*0.054*
Fasting insulin	−0.367	0.111	**−0.565**	0.028*
*Subcutaneous adipocytes ex vivo*				
Basal glucose uptake	**0.480**	0.032*	0.382	0.160
Insulin-stimulated glucose uptake ^a^	**0.611**	0.002*	**0.618**	0.014*
Adipocyte cell size	*0.419*	*0.066*	−0.415	0.124

Table shows Spearman rho-correlation coefficient.

BMI: body mass index. WHR: Waist-to-hip ratio. VAT: visceral adipose tissue. SAT: subcutaneous adipose tissue. HbA1c: glycated haemoglobin. HOMA-IR: Homoeostatic model assessment. AUC: area under curve. NA: not available. Bold*p < 0.05. Italics: 0.05 < *p* < 0.1.

^a^Glucose uptake with 1000 µU/mL of insulin.

### 3.2. Correlation between ESR2 expression and expression of adipogenic, lipid storage, and glucose transport markers in human adipose tissue

Next, we assessed the correlation between *ESR2* expression and adipogenic, lipid storage, and glucose transport markers in SAT (Table 4). *ESR2* expression was positively associated with expression of adipogenic and lipogenic genes (e.g. *LPL*), insulin signalling (e.g. *AKT2*), and the adipokine *ADIPOQ*, and negatively correlated with markers of fatty acid oxidation (e.g. *CPT1A, CPT1B*). This correlation analysis was then validated in a separate cohort (cohort 2) with a wider BMI range (Table 4). In this cohort, we found that all previous correlations were significant, in addition to a positive association between *ESR2* and markers of differentiation (e.g. *PPARG*), lipogenesis (e.g. *ACACA, ELOVL6, FASN, DGAT2*), glucose transport and insulin signalling (e.g. *IRS1, GLUT4*). Also, we assessed associations between *ESR2* expression and markers of cell proliferation (cyclins, CDK’s) and found no significant associations between ESR2 expression and markers of cell proliferation (data not shown). When the male cohort was subdivided by control and T2D subjects, we found that *ESR2* expression had a negative correlation with *ADIPOQ* in control males and a positive correlation with *AKT1* and *AKT2* in males with T2D (data not shown).

**Table 4.** Correlation between *ESR2* mRNA expression and mRNA expression of adipogenic, lipid storage, and glucose transport markers in SAT.

	Cohort 1		Cohort 2	
	n = 20		n = 15	
Gene	Rho	*P*-value	Rho	*P*-value
*Adipogenesis*				
*PPARG*	0.026	0.915	**0.711**	0.003*
*CEBPA*	0.235	0.181	0.306	0.265
*Lipogenesis*				
*LPL*	**0.511**	0.021*	**0.643**	0.010*
*ACACA*	0.402	*0.079*	**0.639**	0.010*
*ELOVL6*	*0.418*	*0.067*	**0.525**	0.044*
*FASN*	0.285	0.224	**0.675**	0.006*
*ACLY*	0.324	0.164	*0.468*	*0.079*
*LPIN2*	0.238	0.313	*0.468*	*0.079*
*DGAT2*	0.236	0.312	**0.525**	0.044*
*Fatty acid oxidation*				
*CPT1A*	**−0.589**	0.006*	−0.297	0.203
*CPT1B*	**−0.502**	0.024*	*−0.479*	*0.079*
*PDK4*	0.224	0.342	0.246	0.376
*ADRB2*	−0.035	0.885	−0.411	0.128
*Lipolysis*				
*HSL*	*0.405*	*0.076*	0.396	0.143
*ATGL*	0.113	0.358	0.171	0.541
*PKA*	0.048	0.840	0.181	0.567
*Adipokines*				
*ADIPOQ*	**0.532**	0.018*	**0.686**	0.005*
*LEP*	0.179	0.450	0.029	0.919
*Glucose transport*				
*AKT1*	−0.041	0.865	0.304	0.271
*AKT2*	**0.494**	0.027*	**0.596**	0.019*
*IRS1*	0.120	0.613	**0.868**	**<0.001**
*GLUT4*	0.194	0.412	**0.639**	**0.010**
*GLUT1*	0.210	0.374	0.161	0.567

Table shows Spearman’s rho correlation coefficient.

*PPARG*: Peroxisome proliferator-activated receptor gamma. *CEBPA*: CCAAT enhancer-binding protein alpha. *LPL*: lipoprotein lipase. *ACACA*: Acetyl-CoA carboxylase 1. *ELOVL6*: Elongation of very long chain fatty acids protein 6. *FASN*: Fatty acid synthase. *ACLY*: ATP Citrate Lyase. *LPIN2*: Lipin 2. *DGAT2*: Diglyceride acyltransferase 2. *CPT1A*: Carnitine palmitoyltransferase I. *CPT1B*: Carnitine Palmitoyltransferase 1B. *PDK4*: Pyruvate Dehydrogenase Kinase 4. *ADRB2*: Adrenoceptor Beta 2. *HSL*: hormone sensitive lipase. *ATGL*: Adipose triglyceride lipase. *PKA*: protein kinase A. *ADIPOQ*: adiponectin. *LEP*: Leptin. *AKT1*: AKT Serine/Threonine Kinase 1. *AKT2*: AKT Serine/Threonine Kinase 2. *IRS1*: Insulin receptor substrate 1. *GLUT4*: Glucose transporter type 4. GLUT1: Glucose transporter type 1. Bold*p < 0.05. Italics: 0.05 < *p* < 0.1.

### 3.3. ESR2 levels in adipose tissue fractions and in vitro differentiated adipocytes

**Figure 1.**
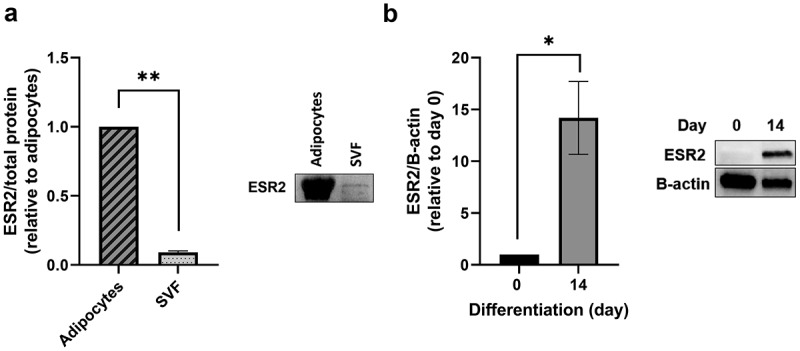
**ESR2 protein levels in SAT cell fractions and *in vitro* differentiated preadipocytes**. Representative immunoblot of ESR2 protein levels of (a) adipocyte and stromal vascular fractions (SVF), respectively, and (b) preadipocytes (d 0) differentiated into adipocytes (d 14). ESR2 protein is adjusted to total protein or to B-actin as indicated (see Methods and Supplementary Figure 1). T-test performed on log-transformed data. n = 3 subjects, cohort 3. Data represent mean ± SEM (relative to adipocyte levels or d 0 levels). *p < 0.05.

We assessed protein levels of ESR2 in mature adipocytes and the SVF of SAT. We found that ESR2 levels were approximately 12-fold higher in adipocytes compared to the SVF fraction (Figure 1(a), p < 0.01). To account for different proportions of adipocytes and SVF cells in SAT, we assessed the total protein content of adipocytes and SVF in whole-adipose samples and estimated that less than 1% of ESR2 is found in the SVF (data not shown). In addition, we have also compared ESR2 protein levels in preadipocytes obtained from SVF culture at the start of differentiation (‘day 0’) and after differentiation into mature adipocytes (‘day 14’) and found that ESR2 levels are 14-fold higher in mature adipocytes than preadipocytes (Figure 1(b), p < 0.05).

### 3.4. Effect of ESR2 knockdown on preadipocyte proliferation, differentiation and lipid accumulation

**Figure 2.**
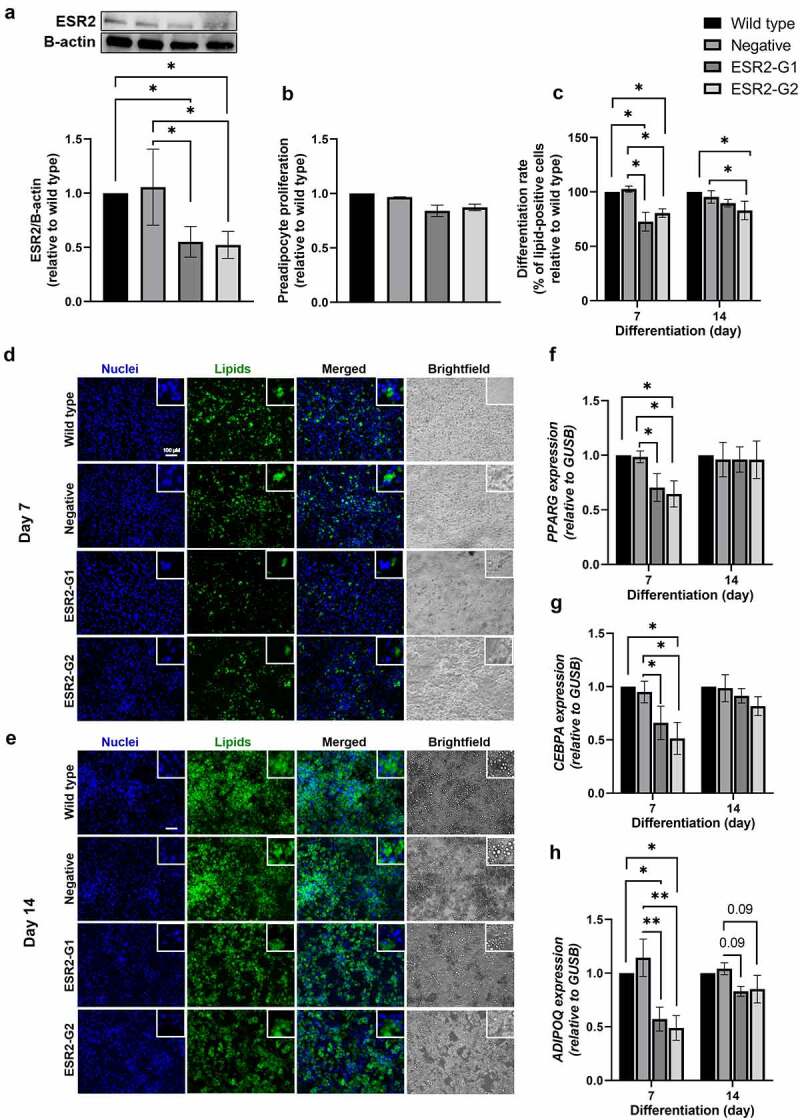
***ESR2* knockdown efficiency, and effects on preadipocyte differentiation rate and expression of adipogenic markers**. (a) Representative immunoblot and quantification of ESR2 protein levels in wild type, negative control and ESR2 knockdown, ESR2-G1 and -G2, cultures on d 14 of differentiation. (b) Preadipocyte proliferation rate of negative control and ESR2 knockdown cultures relative to wild type and (c) differentiation rate, measured as the percentage of lipid-positive cells during differentiation relative to wild type (d 7 and 14). Representative immunofluorescence images of wild type, negative control, ESR2-G1 and -G2 cultures on (d) d 7 and (e) d 14 of differentiation, stained with Hoechst nuclear stain (Nuclei, blue), BODIPY lipid stain (Lipids, green), both combined, shown as Merged, as well as corresponding Brightfield images for (20X magnification segments compared to images). Scale bar: 100 μM. (f) *PPARG*, (g) *CEBPA* and (h) *ADIPOQ* gene expression on d 7 and 14 of differentiation. n = 4–6 (from cohort 3), except for preadipocyte proliferation, n = 3. Data represent mean ± SEM. *p < 0.05, **p < 0.01.

*ESR2* knockdown efficiency in primary human preadipocytes was confirmed at the protein level by western blot, where ESR2 levels were lower by approximately 40–50% in the ESR2-G1 and ESR2-G2 cultures compared to wild type and negative cultures (Figure 2(a)). In addition, guide editing efficiencies were verified in SGBS preadipocytes and were found to be approximately 65% efficient (Supplementary Figure 2). Both *ESR1* and *GPER1* expressions were unaffected by *ESR2* knockdown (data not shown). We found no significant difference between cell proliferation of negative and knockdown cultures (Figure 2(b), p > 0.05).

**Figure 3.**
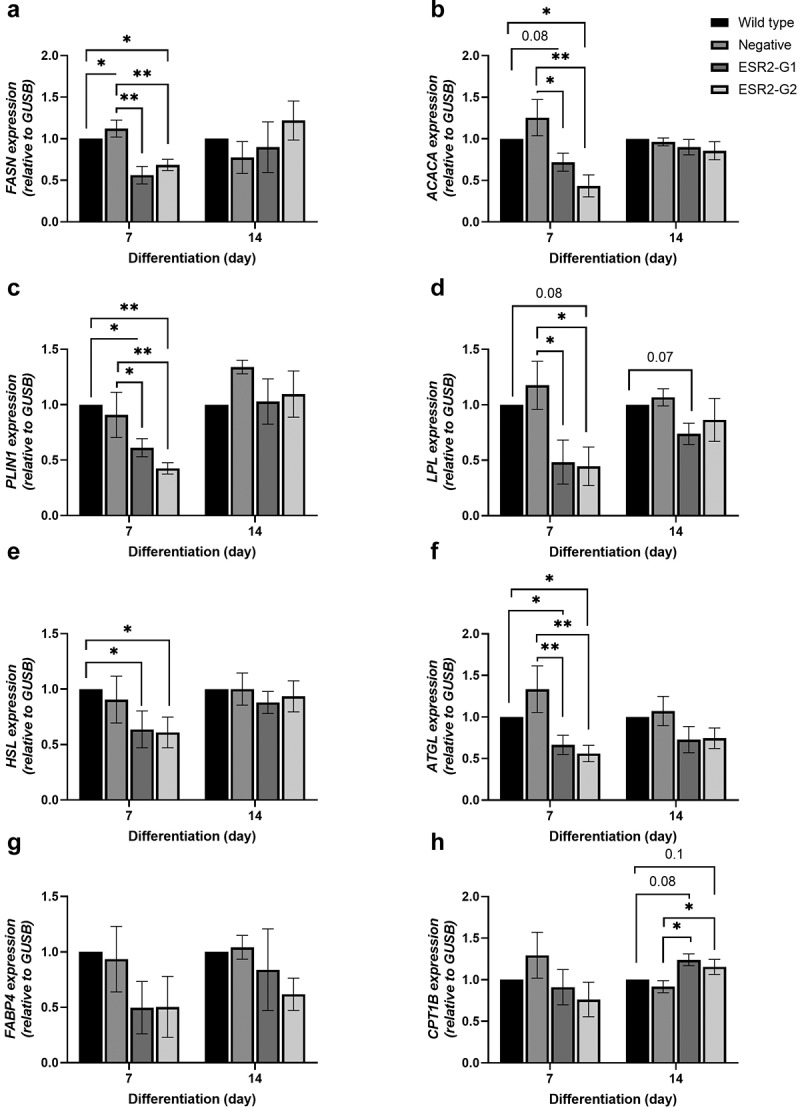
**Expression of lipogenic and lipolytic markers during preadipocyte differentiation**. Gene expression levels of (a) *FASN*, (b) *ACACA*, (c) *PLIN1*, (d) *LPL*, (e) *HSL*, (f) *ATGL*, (g) *FABP4*, and (h) *CPT1B* on d 7 and d 14 of preadipocyte differentiation. Data are relative to wild type. n = 4–6, from cohort 3. Data represent mean ± SEM. *p < 0.05, **p < 0.01.

To assess preadipocyte differentiation, wild type and knockdown cultures were imaged and quantified on d 7 and 14 of differentiation (Figure 2(c-e)). The differentiation rate, measured as the percentage of lipid-positive cells, was reduced by approximately 20–30% on d 7 and 10–20% on d 14 in knockdown cultures compared to wild type and negative cultures (Figure 2(c), p < 0.05). On d 7, ESR2 knockdown resulted in a significant reduction in the expression of markers of preadipocyte differentiation, including *PPARG, CEBPA, ADIPOQ* (figure 2(f-h), p < 0.05), compared to wild type and negative cultures. *ESR2* knockdown resulted in a reduction in the expression of lipogenic markers *FASN, ACACA, PLIN1, and LPL* (Figure 3(a-d), p < 0.05) and the lipolytic markers *HSL, ATGL* (Figure 3(e and f), p < 0.05) compared to wild type and negative cultures. *FABP4* was not significantly different on d 7; however, there was a trend for reduction (Figure 3(g), p > 0.05). *CPT1B*, a marker of fatty acid oxidation, was not significantly different in ESR2 knockdowns compared to wild type and negative cultures on d 7 of differentiation, although there was a significant increase on d 14 of differentiation, compared to negative culture (Figure 3(h), p < 0.05). On d 14, adipogenic genes in *ESR2* knockdown cultures were not significantly different compared to wild type and negative cultures (Figure 3(a-g), p > 0.05).

### 3.5. Effect of ESR2 knockdown on adipocyte glucose uptake

**Figure 4.**
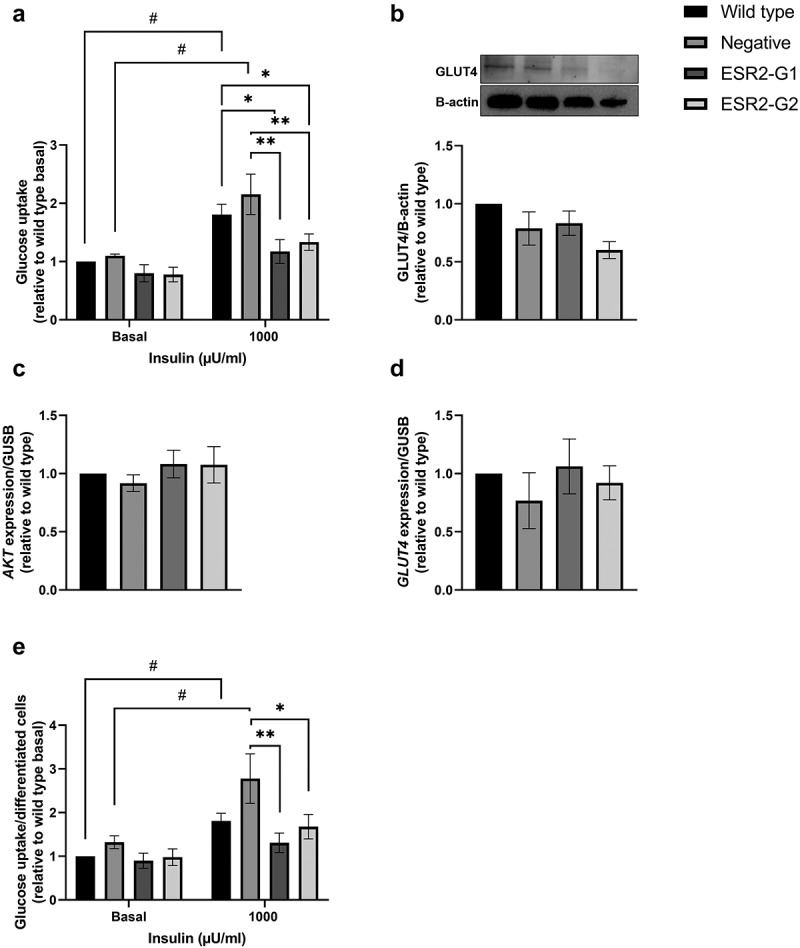
**Effect of *ESR2* knockdown on glucose uptake**. (a) Basal and insulin-stimulated glucose uptake in wild type, negative control and ESR2-G1 and -G2 knockdown cultures on d 14 of differentiation (normalized for protein and relative to wild type basal). (b-c) GLUT4 protein and mRNA and (d) *AKT2* mRNA expression on d 14 of differentiation. (e) Glucose uptake normalized for number of differentiated cells in wild type, negative control and ESR2 knockdown cultures. n = 3–4, cohort 3. Data represent mean ± SEM. *p < 0.05, **p < 0.01. #*p* < 0.05: between groups.

Adipocyte glucose uptake was measured on d 14 of preadipocyte differentiation. *ESR2* knockdown resulted in a reduction in insulin-stimulated glucose uptake when compared to wild type (p < 0.05) and negative (p < 0.01) (Figure 4(a)). The insulin-stimulated increase in glucose uptake was significantly higher than basal glucose uptake in wild type and negative cultures, but not *ESR2* knockdown cultures (Figure 4(a), p < 0.05). Next, we measured gene and protein levels of the glucose transporter GLUT4. *ESR2* knockdown resulted in a nominal reduction in GLUT4 protein levels (Figure 3(b)). There was no difference in *GLUT4* and *AKT* expression in knockdown cultures compared to wild type and negative (Figure 4(c and d)). To determine whether reduced glucose uptake was due to a reduction in preadipocyte differentiation, glucose uptake was normalized to number of differentiated cells. We found that glucose uptake was significantly reduced in knockdown cultures compared to negative when normalized to differentiation rate (Figure 4(e)). In addition, we selected the most effective guide RNA (ESR2-G1) to validate the functional effects of knockdown on glucose metabolism in the human SGBS adipocyte cell line, and we found comparable results to data found in human primary preadipocytes (Supplementary Figure 2).

## 4. Discussion

In this study, we show that *ESR2* expression in adipose tissue from females is negatively correlated with markers of central obesity and fatty acid oxidation markers, and positively correlated to expression markers of lipogenesis and *ex vivo* adipocyte glucose uptake. To assess the functional significance of *ESR2* in adipose tissue, we performed *ESR2* knockdowns in preadipocytes isolated from SAT from females. We found that *ESR2* knockdown reduced preadipocyte differentiation into adipocytes and glucose uptake. These data suggest that *ESR2* may have an important role in adipocyte lipid and glucose metabolism.

Changes in *ESR2* expression have been associated with altered fat distribution and changes in adipose tissue phenotype [7,12,23]. Here, we observed that *ESR2* expression in SAT from females was negatively associated with markers of central obesity such as WHR and VAT volume. It has been shown that obesity-associated systemic factors reduce *ESR2* expression, which may function as a compensatory mechanism to limit lipogenesis and lipolysis in adipose tissue [24]. In addition, *ESR2* SNPs have been implicated in obesity [3,23]. Interestingly, however, we found that *ESR2* expression was positively correlated with markers of lipogenesis. Our findings are in accordance with other studies that have shown that postmenopausal women, who are known to express high levels of *ESR2* [3], display more adipocyte lipid storage [25] and have larger adipocyte cell size compared to premenopausal women [11]. Also, it is possible that *ESR2* promotes the accumulation of fat into subcutaneous adipose tissue and limits accumulation in visceral adipose tissue, as we observed a negative association between *ESR2* levels and VAT volume. This may function as a protective mechanism to promote a healthy obesity phenotype. Alternatively, changes in adipose tissue morphology and redistribution may lead to changes in ESR2 levels. Moreover, ovariectomized mice, a model for the postmenopausal state, have an upregulation of lipogenic genes and a downregulation of markers of fatty acid oxidation [26]. This is in agreement with our data, where we demonstrate that high *ESR2* expression was positively correlated with lipogenic markers, but negatively correlated with fatty acid oxidation markers. Fatty acid oxidation is the largest contributor to ATP production, and reduced fatty acid oxidation leads to the accumulation of lipids, which may then result in insulin resistance [26]. It is possible that higher expression of *ESR2*, such as in postmenopausal women, may contribute to an upregulation of fatty acid storage and reduced fatty acid oxidation.

We did not observe differential expression of *ESR2* levels between control and T2D subjects. Furthermore, when associations between expression of *ESR2* and markers of obesity and insulin resistance were assessed in control subjects and metformin-treated patients with T2D, few correlations were significant and only in males. However, due to the small sample size, major conclusions cannot be made from this. It is possible that metformin may contribute to findings in T2D patients, and in most T2D studies, a large proportion of patients are on metformin treatment. Future studies with larger cohorts are important to investigate the effect of metformin treatment on ESR2 expression and its associations with obesity and insulin resistance.

Since *ESR2* expression in adipose tissue is associated with markers of lipid and glucose metabolism, and levels are increased after menopause [3], we assessed the functional role of *ESR2* on adipocyte lipid and glucose metabolism. We found that in knockdown cultures, preadipocytes had a reduced differentiation on both d 7 and 14. This corresponded to a reduction in expression of lipogenic markers (e.g. *FASN*), differentiation markers (e.g. *PPARG*), and lipolytic markers (e.g. *HSL*), primarily on d 7 of differentiation. This suggests that low *ESR2* in subcutaneous adipocytes from females may be associated with reduced lipid storage in SAT. The mechanism of ESR2 in adipocyte differentiation and lipid storage was not addressed in our study, but it is known that ESR2 functions as a transcription factor and can regulate the expression of oestrogen-responsive genes [1]. In a genome-wide analysis, enriched motifs in oestrogen receptor binding sites of the genome and several adjacent genes were identified, one of which is CEBPA, a major regulator of adipogenesis [27]. ESR2 activation may regulate transcription of these factors, thus altering adipocyte differentiation and lipid storage. Postmenopausal women, who have higher expression of *ESR2*, are known to have an increased tendency to store triglycerides in SAT compared to premenopausal women, which is associated with lipogenesis [3,25]. In addition, in postmenopausal adipocytes, acyl-coA synthetase and diglyceride acyltransferase activity has been shown to be correlated to FFA storage rates [25], and in our study, we found a positive correlation between *ESR2* expression and *ACACA* and *DGAT2*. Together, this suggests higher subcutaneous *ESR2* expression, such as after menopause, may contribute to increased lipid storage in SAT.

Some studies have suggested a negative crosstalk between *ESR2* and *PPARG* expression [28,29]. These studies are in contrast to our study, where we found that *ESR2* knockdown resulted in a transient reduction in *PPARG*. In accordance with our study, 30, showed that in male mice, the selective *ESR2* agonist provokes redistribution of fat mass, with increased *PPARG* expression and lipid storage in SAT [30]. These differences may be due to different factors such as species used and *in vivo* versus *in vitro* models. Most studies have investigated the role of *ESR2* in cell lines [29], which does not account for possible differences in *ESR2* expression and function in humans. In this study, we investigate the role of *ESR2* in primary preadipocytes from females, and whether these effects on differentiation occur in males is unknown. Future studies are needed to compare differences in *ESR2* function on preadipocyte differentiation.

To determine the effects of *ESR2* on adipocyte glucose metabolism, glucose uptake capacity and GLUT4 levels were measured on d 14 of adipocyte differentiation, when the cells have reached functional maturity. We found that in *ESR2* knockdown cultures, insulin-stimulated glucose uptake was reduced, which corresponded to a nominal reduction in GLUT4. It is generally believed that *ESR2* has a repressive role in *GLUT4* expression [31]. A recent study showed that hyperactivity of ESR2 leads to the repression of GLUT4 expression, which may have a diabetogenic effect [32]. In contrast, we show that ESR2 knockdown results in reduced glucose uptake in knockdown cells. The contrasting data may have several explanations. First, the experimental model differs between the studies, with the study by Laurindo et al. being performed in differentiating murine 3T3-L1 cells incubated for 24 h with agonists/antagonists, whereas our study includes ESR2 knockdown in human preadipocytes isolated from SAT from women. Second, there may be differences between knocking down ESR2 vs. activation of the receptor, such as the differences in the ESR1:ESR2 ratio, as previously shown [3]. Future studies performing ESR2 knockdowns on mature adipocytes are needed to determine the effects of ESR2 independent of altered adipocyte differentiation. Ovariectomized mice with primarily *ESR2* signalling (*ESR1* knockout) have improved insulin sensitivity [7,33]. In addition, the phosphorylation of Akt, a marker of insulin sensitivity, was increased in ovariectomized *ESR1* (primarily *ESR2* signalling) knockout mice. *ESR2* has been shown to mediate Akt activation [34]. In our study, we found a positive association between *ESR2* and *AKT* expression in females. Together, this supports the hypothesis that *ESR2* may facilitate glucose transport and increased *ESR2* in SAT after menopause may function to improve glucose uptake [7].

This study has some limitations. We previously found differential expression of *ESR2* in SAT compared to VAT [3]. Therefore, functional studies on the role of *ESR2* in visceral adipose tissue are warranted. This study achieved only a 40–50% knockdown in SVF-derived preadipocytes. Since these cells only retain the capacity to proliferate or differentiate for a limited number of passages [35], it is not feasible to do clonal isolation for a large experimental setup. Future studies are warranted in optimization to achieve higher *ESR2* knockdown in human preadipocytes. This study assesses the percentage of lipid-positive cells in differentiating preadipocyte cultures. However, we do not have data on *ESR2* levels in individual cells and thus cannot assess the correlation between *ESR2* expression of single cells and lipid content. Also, we investigated the role of *ESR2* by performing knockdowns *in vitro*, which does not take into account the surrounding stroma *in vivo*. It is also important to highlight that various factors are used in the in vitro setting to induce preadipocyte differentiation, including T3. Recently, T3 has been shown to stimulate brown adipogenesis; thus, human preadipocytes differentiated *in vitro* may exhibit some characteristics of brown adipocytes [36]. Future studies should investigate the differential role of *ESR2* on preadipocyte differentiation in the context of different clinical characteristics (e.g. comparing preadipocytes from subjects with varying BMI) and further understand the changes that occur during differentiation. In this study, cells were differentiated in the absence of oestrogen. Future studies are of interest to investigate the role of oestrogen treatment throughout preadipocyte differentiation.

## 5. Conclusion

In this study, we assess associations between *ESR2* expression in SAT with clinical characteristics and markers of adipocyte metabolismin women. We found that *ESR2* expression was negatively correlated with markers of central obesity and positively correlated with markers of lipid storage and glucose metabolism. In addition, we found that *ESR2* knockdown in human subcutaneous preadipocytes from women result in reduced preadipocyte differentiation and glucose uptake. Our results suggest that *ESR2* deficiency impairs human adipocyte differentiation, glucose uptake and lipid storage, and this can contribute to less energy partioning to SAT and thus body fat redistribution towards VAT. This provides insight into a potential molecular target to promote a healthy obesity phenotype.

## Supplementary Material

Supplemental Material

## Data Availability

Some or all datasets generated during and/or analyzed during the current study are not publicly available but are available from the corresponding author on reasonable request.
