# Supplementary material for: ESR2 expression in subcutaneous adipose tissue is related to body fat distribution in women, and knockdown impairs preadipocyte differentiation
Source: Adipocyte. 2022 Aug 17;11(1):434–47. doi: 10.1080/21623945.2022.2102116 (PMC9387337; doi:10.1080/21623945.2022.2102116)
Supplement: Supplemental Material [file KADI_A_2102116_SM2370.docx]

**Supplementary information**

**Supplementary methods**

**SGBS cells culturing conditions**

SGBS cells, a kind gift from Dr. Martin Wabitsch (University of Ulm, Ulm, Germany), were cultured in Dulbecco’s modified Eagle’s medium (DMEM)/Ham’s F12 medium supplemented with 33 μM biotin (Sigma), 17 μM pantothenate (Sigma), Anti-anti (100 U/mL of penicillin, 100 µg/mL of streptomycin, 2.5 μg/mL Amphotericin B; Gibco) and non-heat inactivated 10 % fetal calf serum (SGBS medium) under standard laboratory culturing conditions (37 °C, and 5 % CO2) as described before (Fischer-Posovszky et al 2008).

**CRISPR-Cas9 transfections**

In order to knock down the ESR2 gene in human preadipocyte cells, we used CRISPR-Cas9 genome editing. sgRNAs were designed using the online tool https://chopchop.cbu.uib.no/ (2021-03-01) (Labon et al. 2019) (Table S1) and evaluated by standard guide RNA design considerations. The efficiency of four different sgRNAS were assessed in the SGBS cell line in order to select the two most efficient guides for use in primary human preadipocytes experiments. CRISPR-Cas9 transfections were performed as according to the procedure in Kamble et al. 2019. In short, the cells were electroporated with a Neon® Transfection system and The Neon® Transfection System 10 µL Kit (Thermo Fisher) as per the manufacturer’s guidelines. Chemically modified sgRNA (9.3 pmol/reaction) and TrueCutTM Cas9 protein v2 (6 pmol/reaction) (both from Thermo Fisher) were mixed and incubated for 15 min in room temperature to form a RNP complex. 60 000 cells were used per electroporation reaction. After transfection, SGBS cells were cultured in SGBS medium without antibiotics. After 48 hours, medium was supplemented with Anti-anti for continued culturing.

**Assessment of genomic mutation efficiency using Sanger sequencing.**

For each transfection experiment, cells from wild type, negative control, and ESR2 gene-edited cultures were collected for genomic DNA extraction and subsequent Sanger sequencing to assess editing efficiency at passage three post-transfection. Genomic DNA was extracted using the DNeasy Blood and Tissue kit (Qiagen). The CRISPR/Cas9 target sequences were PCR amplified using 20 ng of genomic DNA, Dream Taq Hot Start polymerase (Thermo Fisher) and target-specific primers, amplifying about 600 to 1300 bp around the cut site (Table S1). PCR conditions were: 3 min at 95 °C, followed by two touchdown cycles of 30 s at 95 °C, 20 s at 65 °C, 64 °C, 63 °C, 62 °C, 61 °C and 30 s at 72 °C and ending with 25 cycles of 30 s at 95 °C, 20 s at 60 °C and 30 s at 72 °C. PCR products were Sanger sequenced in both directions using the target-specific primers. Chromatograms were analysed using the online tool ICE (<https://ice.synthego.com/#/>). The highest efficiency was obtained by sgRNA ESR2-G1 and ESR2-G2 (Table S1 and Figure S2).

**References**

Fischer-Posovszky, P., Newell, F. S., Wabitsch, M., & Tornqvist, H. (2008). Human SGBS Cells - a Unique Tool for Studies of Human Fat Cell Biology. Obesity Facts, 1(4), 184-189.

Kamble, P. G., Hetty, S., Vranic, M., Almby, K., Castillejo-López, C., Abalo, X. M, Pereira, MJ Eriksson, J. W. (2020). Proof-of-concept for CRISPR/Cas9 gene editing in human preadipocytes: Deletion of FKBP5 and PPARG and effects on adipocyte differentiation and metabolism. Sci Rep, 10(1), 10565.

Labun, K., Montague, T. G., Krause, M., Torres Cleuren, Y. N., Tjeldnes, H., & Valen, E. (2019) CHOPCHOP v3: expanding the CRISPR web toolbox beyond genome editing. Nucleic Acids Research.

**Supplementary Tables and Figures**

**Table S1.** CRISPR-Cas9 sgRNA design and primers for genomic sequencing of cut sites

PAM: Protospacer adjacent motif. DBS: Double stranded break. Forwards sequencing primer: Primer seq FW. Reverse sequencing primer: Primer seq RV..

^a^ N=1-3.

**Table S2. Anthropometric and clinical characteristics of the participating males.**

|  |  |
| --- | --- |
| N | 20 |
| Type 2 Diabetes | 10 |
| Age (years) | 58 ± 11 |
| BMI (kg/m^2^) | 29.7 ± 3.9 |
| WHR | 1.01 ± 0.05 |
| Body fat (%) | N/A |
| Plasma glucose (mmol/L) | 7.6 ± 1.8 |
| HbA1c (mmol/mol) | 44.8 ± 10.9 |
| Serum insulin (mU/L) | 13.0 ± 5.7 |
| HOMA-IR | 4.4 ± 2.5 |
| Total cholesterol (mmol/L) | 5.2 ± 1.4 |
| Plasma HDL-cholesterol (mmol/L) | 1.2± 0.3 |
| Plasma triglycerides (mmol/L) | 1.7 ± 0.6 |
| Plasma LDL-cholesterol (mmol/L) | 3.4 ± 1.1 |

Data represent mean ± SD. Blood chemistry is fasting.

BMI: body mass index. WHR: Waist-to-hip ratio. HbA1c: glycated hemoglobin. HOMA-IR: Homeostatic model assessment. HDL: high density lipoprotein. LDL: low density lipoprotein.

**Supplementary Figure 1**

**Figure S1. Total protein measurements in 10** **μg of adipocytes and SVF cell extracts.** Quantification of total protein and representative stain-free blot image of adipocytes and SVF from one subject (N=3). Data represent mean ± SEM.

**Supplementary Figure 2**

**
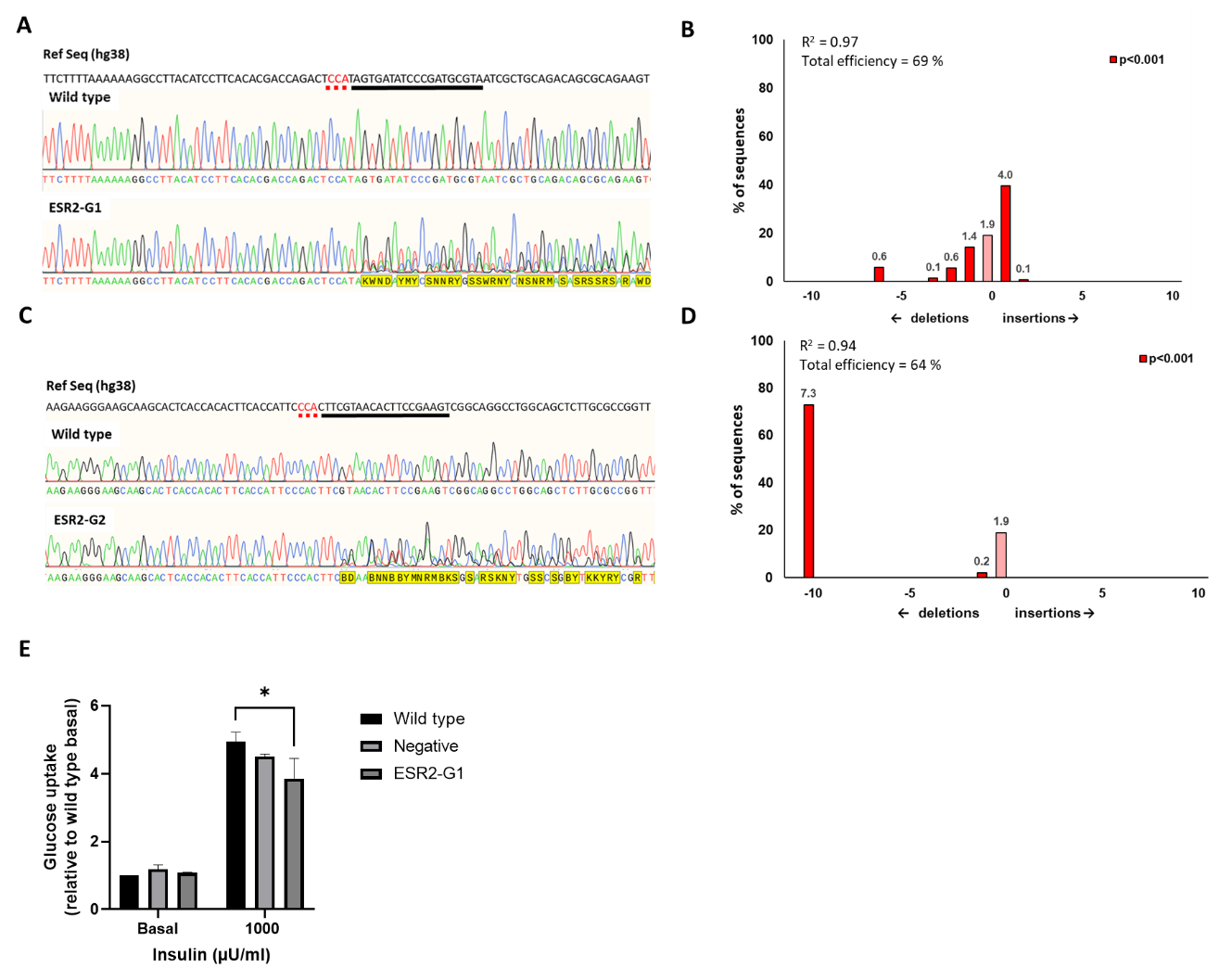
**

**Figure S2. Assessment of DNA level mutation efficiency and glucose uptake phenotype assessment of SGBS ESR2-KD cultures.** Representative Sanger sequencing chromatograms showing the reference (human genome assembly GRCh38; hg38), wild type and edited sequences for the two most efficient CRISPR-Cas9 sgRNA guides targeting the ESR2 gene, (A) ESR2-G1 and C) ESR2-G2, and size distributions of insertions (plus) and deletions (minus) for (B) ESR2-G1 and (D) ESR2-G2 in the entire population of edited cells. (E) Basal and insulin-stimulated glucose uptake in wild type and knockout cultures on day 14 of differentiation (n=3), *p<0.05. R^2^ is the correlation coefficient calculated to assess the goodness of fit of the model and Total efficiency is the percentage editing efficiency in the pool of cells sequenced. Black bar underlines sgRNA sequence and red dotted bar the PAM sequence. Cutoff for statistical significance of indels is p<0.001.
